# Celecoxib suppresses autophagy and enhances cytotoxicity of imatinib in imatinib-resistant chronic myeloid leukemia cells

**DOI:** 10.1186/s12967-016-1012-8

**Published:** 2016-09-20

**Authors:** Ying Lu, Ling-Ling Liu, Shou-Sheng Liu, Zhi-Gang Fang, Yong Zou, Xu-Bin Deng, Zi-Jie Long, Quentin Liu, Dong-Jun Lin

**Affiliations:** 1 Department of Blood Transfusion, the Third Affiliated Hospital, Sun Yat-sen University, Guangzhou, China; 2 Department of Hematology, the Third Affiliated Hospital, Sun Yat-sen University, Guangzhou, China; 3 State Key Laboratory of Oncology in South China, Department of VIP Region, Collaborative Innovation Center for Cancer Medicine, Sun Yat-sen University Cancer Center, Guangzhou, China; 4 Cancer Hospital of Guangzhou Medical University, Guangzhou, China; 5 Institute of Hematology, Sun Yat-sen University, Guangzhou, China

**Keywords:** Celecoxib, Imatinib, Autophagy, CML

## Abstract

**Background:**

Chronic myelogenous leukemia (CML) is a hematological stem cell disorder. Tyrosine kinase inhibitors (TKIs) are the standard treatments for CML, but a number of patients fail to respond effectively due to gene mutations. Celecoxib, a cyclooxygenase-2 (COX-2) inhibitor, has been shown to have anti-tumor effect on solid tumor whereas the anti-CML effect and its underlying mechanism have not been completely elucidated.

**Methods:**

The cytotoxic effects of celecoxib and/or imatinib were evaluated by MTT assay. Cell cycle distribution was examined by propidium iodide (PI) assay. Apoptosis or necrosis was analyzed by Annexin-V/PI, Hoechst 33342 staining and Western blot assays. Autophagy suppression effect of celecoxib was examined by Western blot and LysoTracker probe labelling. Lysosensor probe labelling was used to detect the effect of celecoxib on the lysosomal function.

**Results:**

In this study, we found that celecoxib had therapy efficacy in KBM5 and imatinib-resistant KBM5-T315I CML cell lines. Celecoxib caused significant cytotoxic effect in both cell lines, especially in KBM5-T315I cells exposed to celecoxib for 72 h. Moreover, celecoxib induced necrosis and apoptosis while inhibited autophagy in CML cell lines and patient samples. Furthermore, this study demonstrated that celecoxib prevented the autophagic flux by inhibiting lysosome function. Celecoxib was tested in combination with imatinib, demonstrating that celecoxib could strengthen the cytotoxicity of imatinib in imatinib-resistant CML cells.

**Conclusions:**

These findings showed that celecoxib had therapy efficacy on CML cells. And it is first time to demonstrate that celecoxib is an autophagy suppresser and a combination of celecoxib and imatinib might be a promising new therapeutic strategy for imatinib-resistant CML cells.

**Electronic supplementary material:**

The online version of this article (doi:10.1186/s12967-016-1012-8) contains supplementary material, which is available to authorized users.

## Background

Chronic myelogenous leukemia (CML) is a hematological stem cell disorder responsible for 15–20 % of newly diagnosed leukemia in adults. More than 90 % of CML patients are characterized by the Philadelphia chromosome carrying the Bcr-Abl fusion gene [1, 2]. Current therapeutic regimens include treatment by tyrosine kinase inhibitors (TKIs) and allogeneic hematopoietic stem cell transplantation (allo-HSCT) [3, 4]. Imatinib, recommended as a frontline therapy for chronic phase CML, gives a good clinical response. Unfortunately, some patients, especially in the progressive phase of the disease, show no response or relapse due to resistance. The major mechanism of imatinib resistance is a Bcr-Abl kinase domain mutation [5] and the existence of leukemia stem cells [6]. Allo-HSCT is recommended as an initial treatment for younger patients with HLA-matched donors and is the only curative treatment as of today. However, not all patients are eligible for allo-HSCT. Therefore, it is critical to continue research for novel therapeutic approaches.

Celecoxib is a non-steroidal anti-inflammatory drugs (NSAIDs) and selective cyclooxygenase-2 (COX-2) inhibitor that is used to treat osteoarthritis and rheumatoid arthritis. Recent studies show that celecoxib can regress colon cancer xenografts [7] and enhance the efficacy of chemotherapy [8] and/or radiation treatment [9]. Celecoxib has been approved by the FDA for the treatment of familial adenomatous polyposis (FPA) to reduce the incidence of colon and rectal cancer [10]. Furthermore, its effects on esophagus, liver, breast, lung and prostate cancers have also been confirmed [11–15]. The antitumor effect of celecoxib is associated with cell cycle arrest, apoptosis and autophagy induction [16, 17]. However, there are only few studies on the effects of celecoxib on hematological malignancies. These studies revealed that celecoxib could inhibit the proliferation of lymphoma, acute leukemia or CML cells [18–21]. Celecoxib was reported to have anti-tumor effects on K562 and HL-60 leukemia cells demonstrated by cell-cycle arrest and cell apoptosis and the effects were synergistic with other chemotherapy medicines [19, 21]. However, the molecular mechanism of these anti-tumor effects has not been well elucidated.

Autophagy is a conserved catabolic process where cell constituents are incorporated into autophagosomes that fuse with lysosomes after which the contents are degraded and recycled [22]. The adaptor protein p62 and LC3 are autophagy associated proteins [23, 24]. In tumorgenesis the function of autophagy is complex, it is not always pro-death but can also be pro-survival under conditions of cellular stress [25, 26]. Celecoxib could induce autophagy in solid tumors such as urothelial carcinoma and colorectal cancer and studies revealed that autophagy inhibition enhances cancer cell apoptosis [16, 27]. However, the effect on autophagy of celecoxib in hematopoietic tumors has not been well established.

In this study, we examined the effect of celecoxib on cell proliferation, necrosis, apoptosis and autophagy in CML cell lines KBM5 and KBM5-T315I. The results showed that celecoxib had cytotoxic effect on KBM5 and imatinib-resistant KBM5-T315I cells. Apoptosis and necrosis was induced as reported, however contrary to other reports [16, 17], autophagy was inhibited by celecoxib in the two cell lines. Furthermore, this study found that celecoxib could suppress the autophagic flux by preventing the lysosome function. At last, celecoxib was demonstrated to strengthen the cytotoxicity of imatinib in imatinib-resistant CML cells. So, celecoxib might serve as a new tool to enhance the antitumor activity of conventional therapeutic agents in imatinib-resistant CML cells.

## Methods

### Cell line and primary CML cells

The human leukemia cell lines KBM5 and KBM5-T315I were gifts from Professor Peng Huang (M. D. Anderson Cancer Center, Houston, TX). Six samples of primary CML cells were obtained from patients with newly diagnosed CML (data of patients is shown in Table 1). All patients gave written informed consent for the use of cells for research purposes. In each case, the diagnosis was based on morphological and cytochemical staining and cytogenetic analyses.

**Table 1:** Characteristic of patients

No.	Age/sex	Diagnose	WBC (10^9^/L)	HGB (g/L)	PLT (10^9^/L)	BCR/ABL	Treatment	Time of disease (month)	Survival
P1	43 Y/M	CML-CP	290	72	931	Positive	Imatinib	11	Yes
P2	42 Y/F	CML-CP	282	77	673	Positive	Imatinib	11	Yes
P3	32 Y/F	CML-CP	34	131	505	Positive	Imatinib	10	Yes
P4	33 Y/M	CML-CP	165	85	396	Positive	Dasatinib	10	Yes
P5	27 Y/M	CML-CP	288	68	560	Positive	Imatinib	9	Yes
P6	63 Y/M	CML-AP	320	77	887	Positive	Imatinib	21	No

*P* patient; *Y* year; *M* male; *F* female; *CML*-*CP* chronic myelogenous leukemia-chronic phase; *CML*-*AP* chronic myelogenous leukemia-acute phase; *WBC* white blood cells; *HGB* hemoglobin; *PLT* platelet

### Cell culture

The cells were maintained at 37 °C with 5 % CO_2_ in RPMI-1640 medium supplemented with 10 % fetal bovine serum (FBS). The cell culture media and supplements were purchased from Gibco. For primary CML cells, mononuclear cells (BMMNCs) were isolated by means of Ficoll density gradient centrifugation.

### Reagents and antibodies

Reagents included celecoxib (Pfizer, New York, NY), imatinib (Novartis Pharma, Basel, Switzerland), chloroquine (Sigma, St. Louis, MO). LC3 antibody was purchased from Novus Biologicals (Littleton, CO), SQSTM1/p62 antibody was purchased from Santa Cruz Biotechnology (Dallas, TX). Antibodies against cleaved caspase-3, 4E-BP1, phospho-4E-BP1, mTOR, phospho-mTOR were obtained from Cell Signaling Technology (Danvers, MA). HRP (horseradish peroxidase)-labeled goat anti-rabbit IgG and goat anti-mouse IgG were bought from Protein Tech Group (Chicago, IL). MTT [3-(4,5-dimethylthia-zol-2-yl)-2, 5-diphenyltetrazolium bromide], Hoechst 33342 and propidium iodide (PI) were obtained from Sigma (St. Louis, MO). Annexin V-PI apoptosis detection kit was provided by BD Biosciences Pharmingen (Franklin Lakes, NJ).

### MTT assay

Cell viability was assessed by MTT assay. Cells were seeded in 96-well plates and treated with celecoxib and/or imatinib for 24, 48 or 72 h. Then MTT was added and incubated for 4 h, followed by centrifugation at 1500 rpm for 5 min. Supernatants were removed and the remaining MTT dye was solubilized with 200 μl DMSO. The optical density was measured at 490 nm using a multi-well plate reader (Micro-plate Reader; Bio-Rad, Hercules, CA).

### Cell cycle analysis

Cells were collected and fixed with 70 % ethanol at −20 °C overnight. Then cells were washed three times and stained with a mixture of PI (50 μg/ml), 0.2 % Triton X-100 and RNase inhibitor (100 μg/ml) for 15 min in the dark. Cell cycle analysis was performed using a FACS flow cytometer equipped with Modfit LT for Mac V2.0 software (BD Biosciences, San Jose, CA).

### Hoechst 33342 staining

Nuclear fragmentation was examined by Hoechst 33342. Cells treated with celecoxib for 24 h were stained with Hoechst 33342 (10 μg/ml) for 15 min at 37 °C. Slides were viewed using a fluorescence microscope. Two hundred cells were counted for statistics.

### Apoptosis analysis

According to the instruction, approximately 1 × 10^6^ cells per well were treated with 0, 20, 40, 60 and 80 μM concentrations of celecoxib. Then cells were collected and stained with Annexin V/PI. Flow cytometry was used to analyze the percentage of Annexin V-/PI+ (necrosis), Annexin V+/PI- (early apoptosis) and Annexin V+/PI+ (late apoptosis) cells.

### Western blot analysis

Cells were collected and total protein was isolated with lysis buffer. Equal amounts of protein were subjected to sodium dodecyl sulfate-polyacrylamide gel electrophoresis (SDS-PAGE) and transferred to polyvinylidene difluoride membranes. The membranes were blocked and then incubated with antibodies. Subsequently, the membranes were incubated with a HRP-conjugated secondary antibody at room temperature for 1 h. Blots were detected with an enhanced chemiluminescence reagent (Sigma), according to the manufacturer’s instructions.

### LysoTracker and Lysosensor labelling

Cells were harvested and stained with LysoTracker Green (50 nM, Cat. No. L7526, Invitrogen, Carlsbad,CA), LysoSensor Green (1 µM, Cat. No. L7535, Invitrogen,Carlsbad,CA), and LysoTracker ^®^ Red DND-99 (75 nM, Cat. No. L7528, Invitrogen, Carlsbad, CA) dye for 30 min at 37 °C according to the instructions. Slides were imaged using confocal microscopy (ZEISS, Germany).

### Statistics

All data were presented as mean ± SD of three determinations. One-way ANOVA followed by Bonferroni multiple comparison was performed to assess the differences between two groups under multiple conditions. If the data failed the normality test, the Kruskal–Wallis one-way ANOVA on ranks was used. A value of *p* < 0.05 was considered statistically significant. Jin’s formula was used to evaluate the synergistic effects of drug combinations. Jin’s formula is given as: Q = Ea + b/(Ea + Eb—Ea × Eb). Ea + b represents the cell proliferation inhibition rate of the combined drugs, while Ea and Eb represent the rates for each drug respectively. A value of Q = 0.85–1.15 indicates a simple additive effect, while Q > 1.15 indicates synergism.

## Results

### Cytotoxic effect of celecoxib on human CML cells

**Fig. 1:**
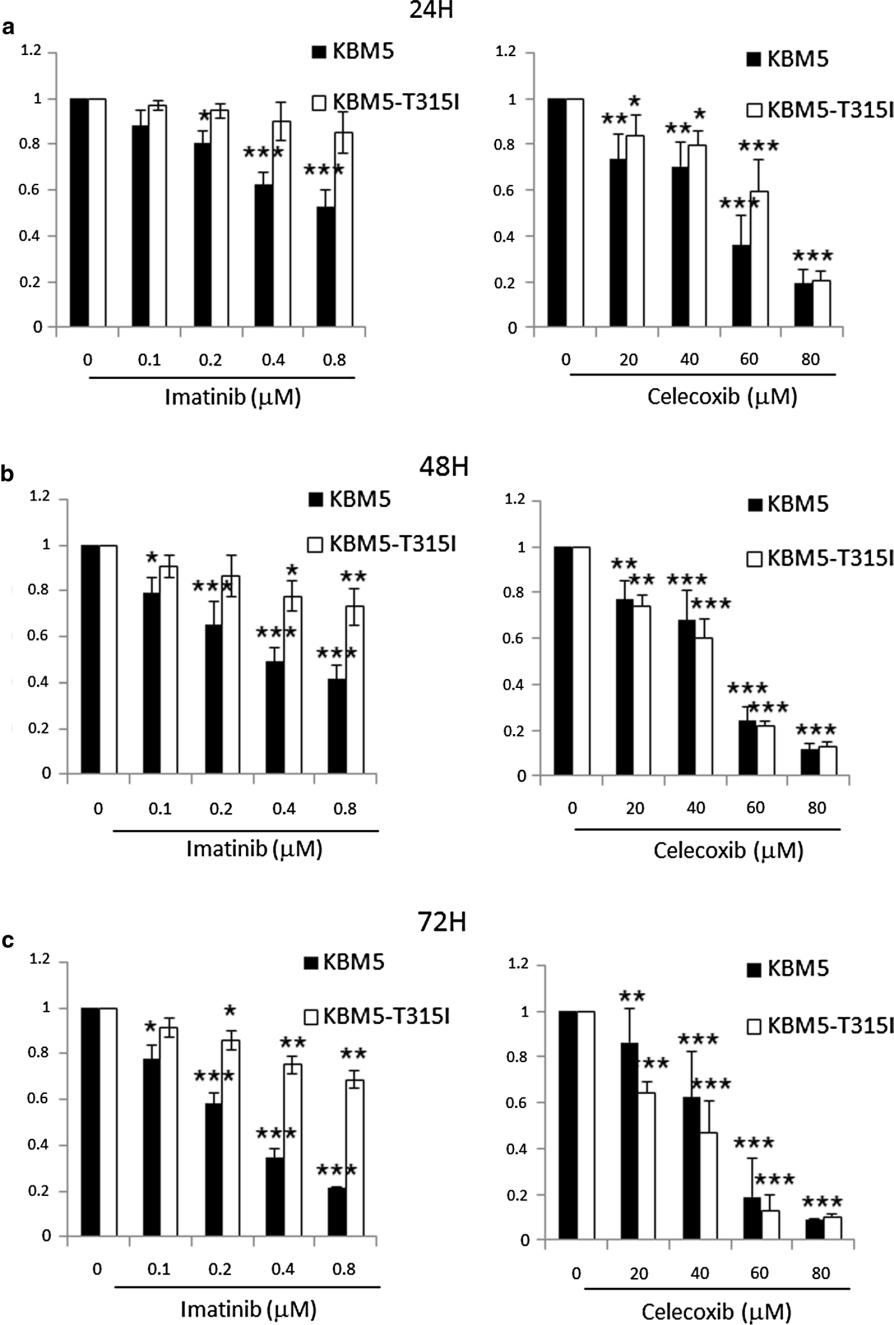
The cytotoxic effect of celecoxib and imatinib on KBM5 and KBM5-T315I cells. **a**–**c** KBM5 and KBM5-T315I cells were treated with 0, 20, 40, 60, 80 μM celecoxib or 0, 0.1, 0.2, 0.4, 0.8 μM imatinib for 24, 48, 72 h, then cell viability was measured by MTT assay. Data were mean ± SD (n = 3). *p < 0.05, **p < 0.01, ***p < 0.001

First, the cytotoxic effect of celecoxib and imatinib on CML cells was measured by MTT assay. Two cell lines were used for this, KBM5 an imatinib-sensitive cell line and KBM5-T315I an imatinib-resistant cell line with a T315I site mutation. They were exposed to 0, 20, 40, 60 and 80 μM celecoxib for 24, 48 and 72 h, respectively. As shown in Fig. 1a–c, both celecoxib and imatinib had cytotoxic effect on the two cell lines in a concentration- and time-dependent manner. The IC50 values of imatinib at 24, 48 and 72 h were 0.8, 0.5 and 0.3 μM for KBM5 cells and 5.7, 3.5 and 1.9 μM for KBM5-T315I cells, which verified imatinib resistance for KBM5-T315I cells. For celecoxib, the IC50 values in KBM5 at 24, 48 and 72 h were 40.4, 39.0 and 39.5 μM and for KBM5-T315I cells were 50.7, 36.5 and 28.5 μM.

**Fig. 2:**
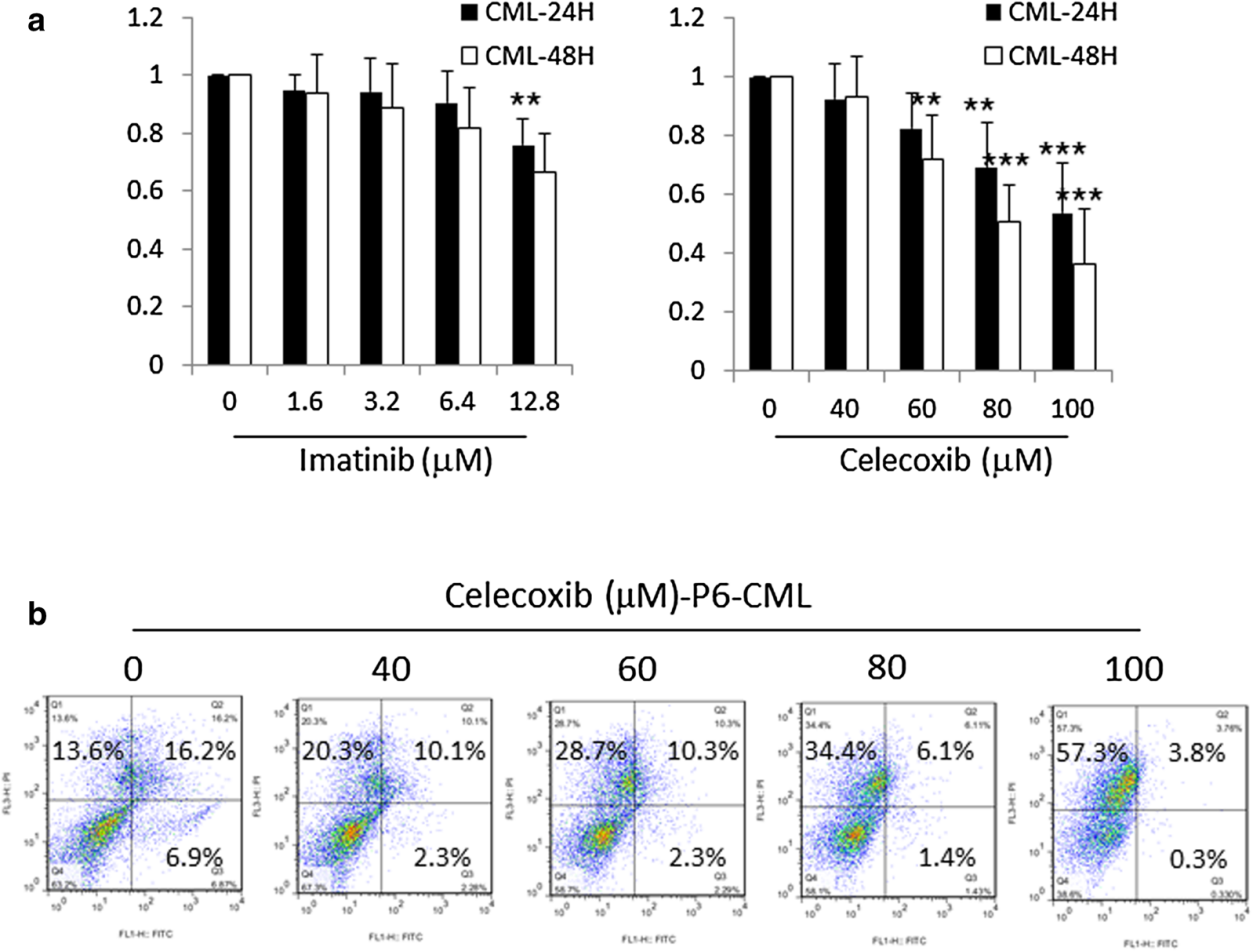
The effect of celecoxib and imatinib on human CML patient samples. **a** Mononuclear cells extracted from human CML patient samples and were treated with 0, 40, 60, 80, 100 μM celecoxib or 0, 1.6, 3.2, 6.4, 12.8 μM imatinib for 24, 48 h, then followed with MTT assay. Data were mean ± SD (n = 5). **p < 0.01, ***p < 0.001. **b** The sixth human CML patient sample was treated with 0, 40, 60, 80, 100 μM celecoxib for 24 h. Annexin V/PI assays was performed by flow cytometry

We next tested the effect of celecoxib on primary CML cells. Mononuclear cells were separated from bone marrow cells or peripheral blood cells of CML patients (data of patients is shown in Table 1). The mononuclear cells were exposed to different concentration of celecoxib for 24 and 48 h. As shown in Fig. 2a, the primary CML cells were sensitive to celecoxib. About 46.3 ± 16.9 and 63.3 ± 18.3 % viability of primary cells were suppressed by 100 μM celecoxib at 24 and 48 h.

### Celecoxib arrests CML cells at cell cycle G1 phase

**Fig. 3:**
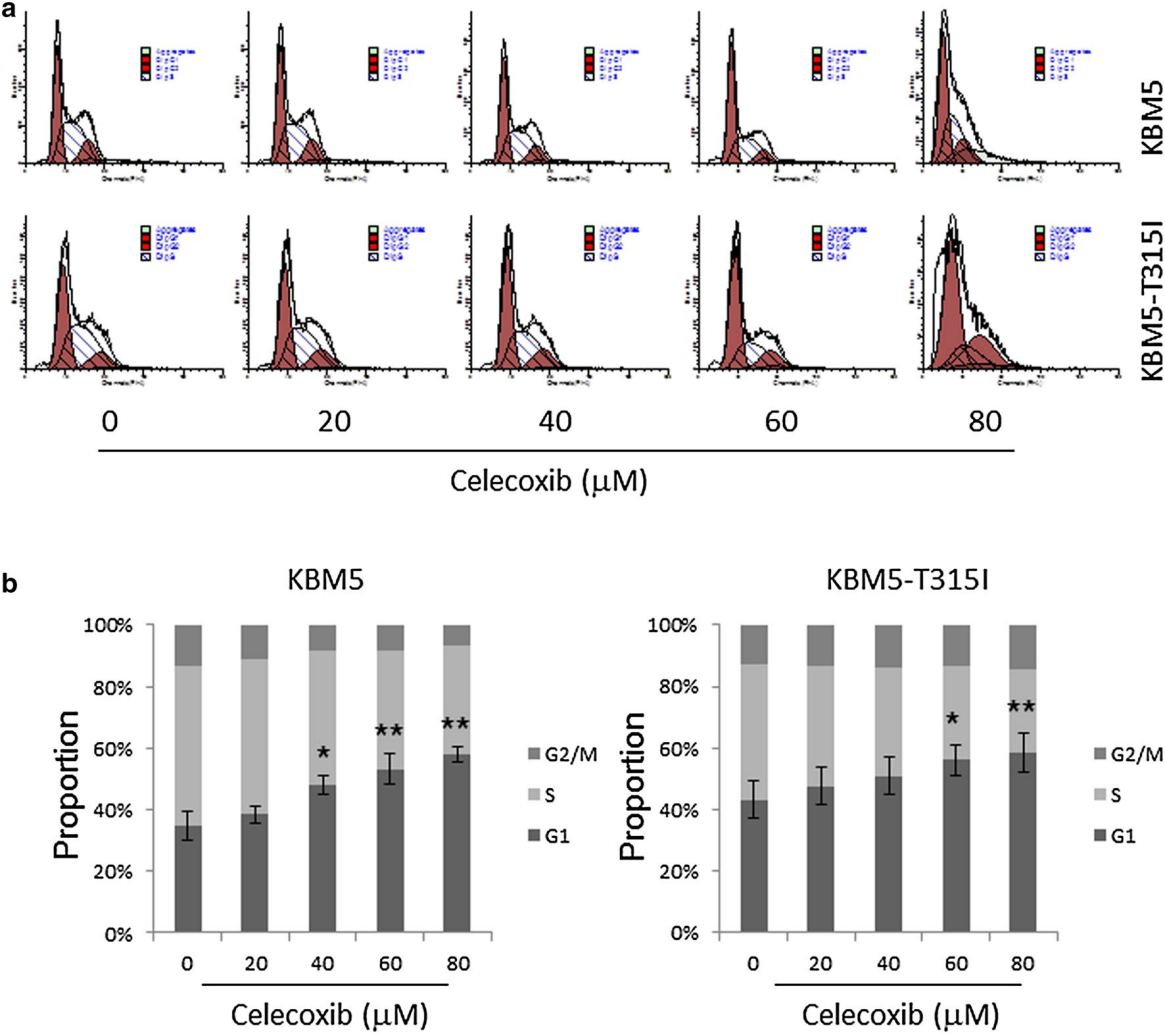
Celecoxib arrests KBM5 and KBM5-T315I cells at cell cycle G1 phase. **a** The two cell lines were treated with 0, 20, 40, 60, 80 μM celecoxib for 24 h. Flow cytometry analysis was conducted for the cell-cycle distribution. **b** The graph showed the percentage of cells in each stage of the cell cycle. Data were mean ± SD (n = 3). *p < 0.05, **p < 0.01

Flow cytometry analysis was used to determine the effect of celecoxib on the cell cycle for KBM5 and KBM5-T315I cells. As shown in Fig. 3a, cells treated with different doses of celecoxib were arrested at G1 phase in a concentration-dependent manner. Treatment of KBM5 cells with 80 μM celecoxib resulted in an increase in the percentage of cells at G1 phase, from 34.9 ± 4.7 to 58.0 ± 2.5 %, with a reduction of cells at S phase (Fig. 3b left) (*p* *<* 0.05). Similar results were found in KBM5-T315I cells where the percentage of cells in the G1 phase went up from 43.2 ± 6.1 to 58.7 ± 6.4 % (Fig. 3b right, *p* *<* 0.05).

### Celecoxib induces apoptosis and necrosis in CML cells

**Fig. 4:**
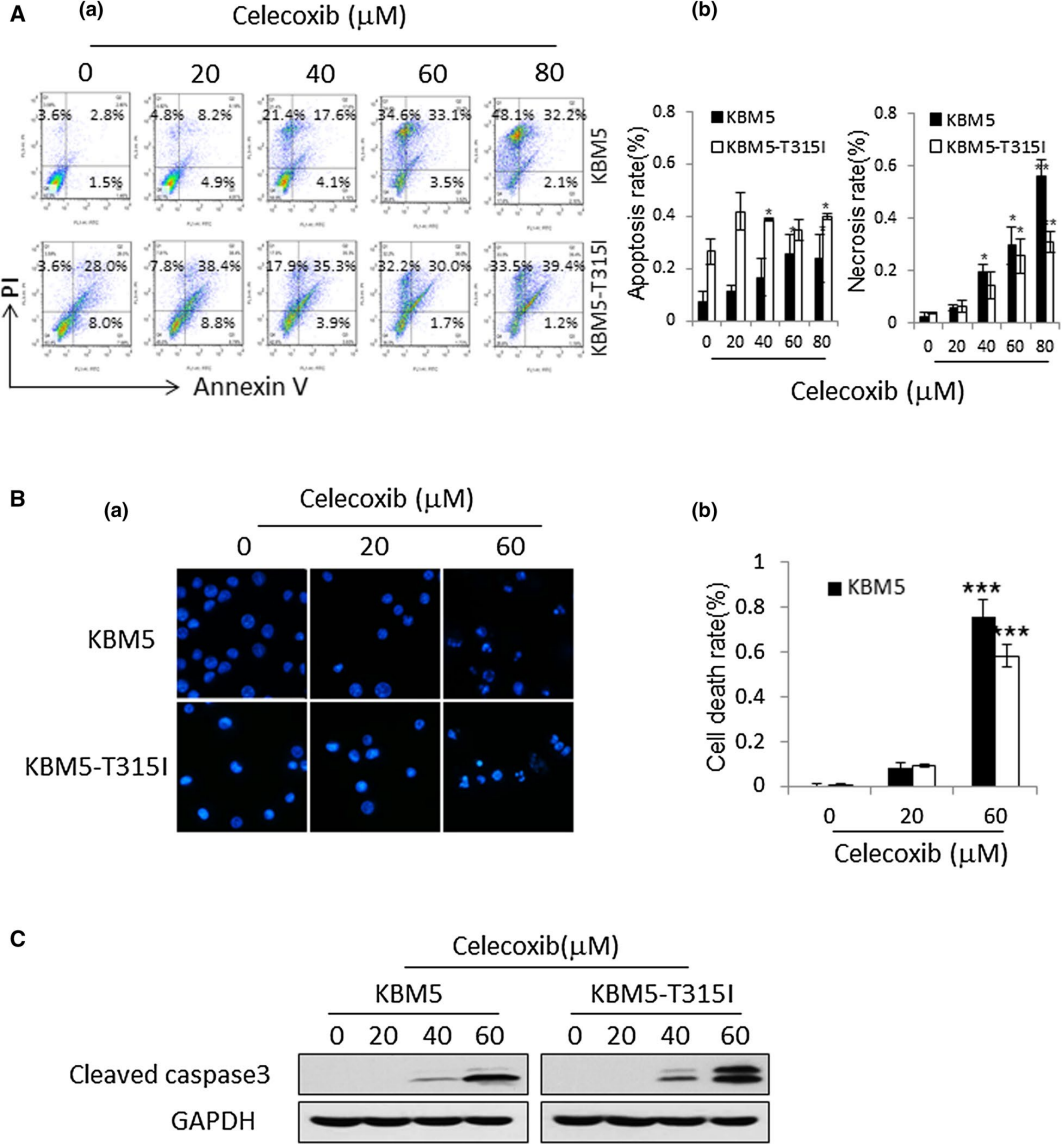
Celecoxib induces apoptosis and necrosis in KBM5 and KBM5-T315I cells. KBM5 and KBM5-T315I cells were treated with different concentrations of celecoxib for 24 h. **A** (*a*) Flow cytometry was used to test the apoptosis and necrosis. (*b*) The *graph* showed the percentage of Annexin V-/PI+ (necrosis) cells in* left*, and showed the Annexin V+/PI- (early apoptosis) and Annexin V+/PI+ (late apoptosis) cells in* right*. **B** (*a*) Hoechst 33342 staining was used to examine the apoptosis cells. (*b*) The graph showed that the percentage of cells with nuclear fragmentation. **C** Western blotting analysis showed cleaved caspase-3 fragment. Data were mean ± SD (n = 3). *p < 0.05, **p < 0.01, ***p < 0.001

To test the ability of celecoxib to induce apoptosis in CML cells, cells were treated with celecoxib for 24 h and tested with flow cytometry. As shown in Fig. 4A (a), the amount of Annexin V+/PI+ (late apoptosis) and Annexin V-/PI+ (necrosis) stained cells were both increased as celecoxib was added to KBM5 and KBM5-T315I cells. When cells were treated with 80 μM celecoxib, the early and late apoptosis rates were 23.8 ± 9.1 % for KBM5 and 39.7 ± 10.6 % for KBM5-T315I cells, respectively [Fig. 4A (b)]. The necrosis rates were 56.2 ± 5.8 % for KBM5 and 30.8 ± 3.7 % for KBM5-T315I when cells treated with 80 μM celecoxib [Fig. 4A (b)]. However, for CML patient 6 cells (p6) treated with celecoxib, the proportion of Annexin V+/PI+ (late apoptosis) stained cells decreased and Annexin V-/PI+ (necrosis) stained cells accumulated (Fig. 2b). Hoechst 33342 dye was used to detect the nuclear morphology with fluorescence microscopy. As shown in Fig. 4B (a), the nuclei of untreated cells appeared round in shape, while celecoxib caused nuclear condensation and simultaneously induced morphological changes in portions of the cells after 24 h. As shown in Fig. 4B (b), 76.1 ± 7.0 % KBM5 cells and 58.5 ± 4.8 % KBM5-T315I cells died when treated with 60 μM celecoxib. Western blot assay showed that the expression of cleaved caspase-3 protein was significantly up-regulated in KBM5 and KMB5-T315I cells treated with 60 μM celecoxib for 24 h (Fig. 4C).

### Celecoxib suppresses autophagy at its late stage in CML cells

**Fig. 5:**
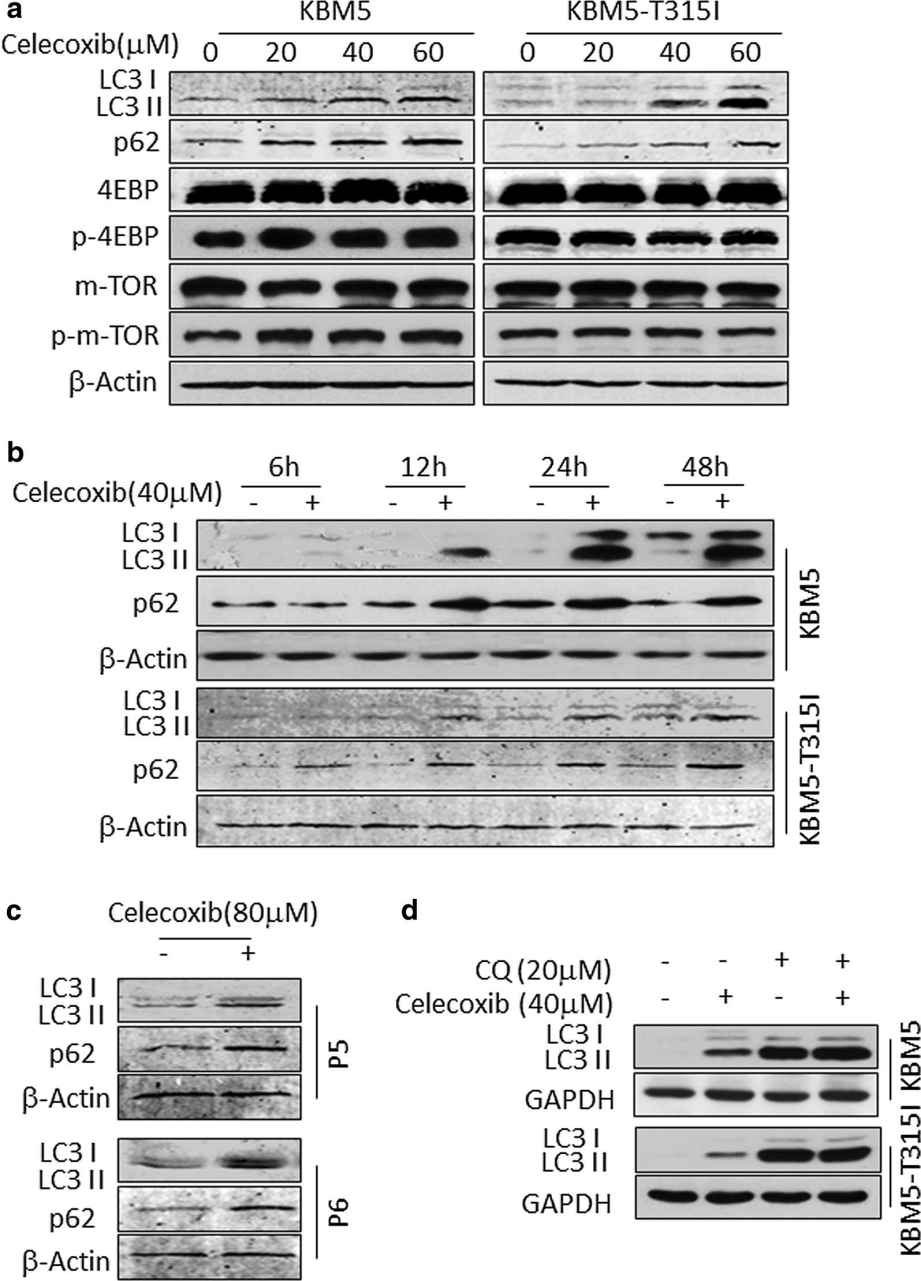
Celecoxib suppresses autophagy in KBM5 and KBM5-T315I cells. **a** KBM5 and KBM5-T315I cells were treated with 0, 20, 40, 60 μM celecoxib for 24 h. **b** The two cell lines were treated with 40 μM celecoxib for 6, 12, 24, 48 h. **c** Two samples for human CML patients were incubated with 80 μM celecoxib for 24 h. **d** KBM5 and KBM5-T315I cells were added with 20 μM CQ and/or 40 μM celecoxib for 24 h. **a**–**d** Cell lysates were subjected to Western blot analysis with indicated antibodies. The results shown are representative of three independent experiments

LC3 and p62 proteins were detected as autophagy associated markers. When autophagy is induced, the ratio of LC3-II to LC3-I protein level increases and the expression of protein p62 reduces due to degradation [28, 29]. As shown in Fig. 5a, when the concentration of celecoxib increased, the ratio LC3-II to LC3-I protein level was raised as well as the expression of protein p62. Moreover, the expression of LC3-II and p62 protein was up-regulated in a time-dependent manner with celecoxib treatment (Fig. 5b). The effect of celecoxib was also confirmed in CML patient samples (Fig. 5c). These results were not identical as the reports [16, 17, 27] that said that celecoxib could induce autophagy.

**Fig. 6:**
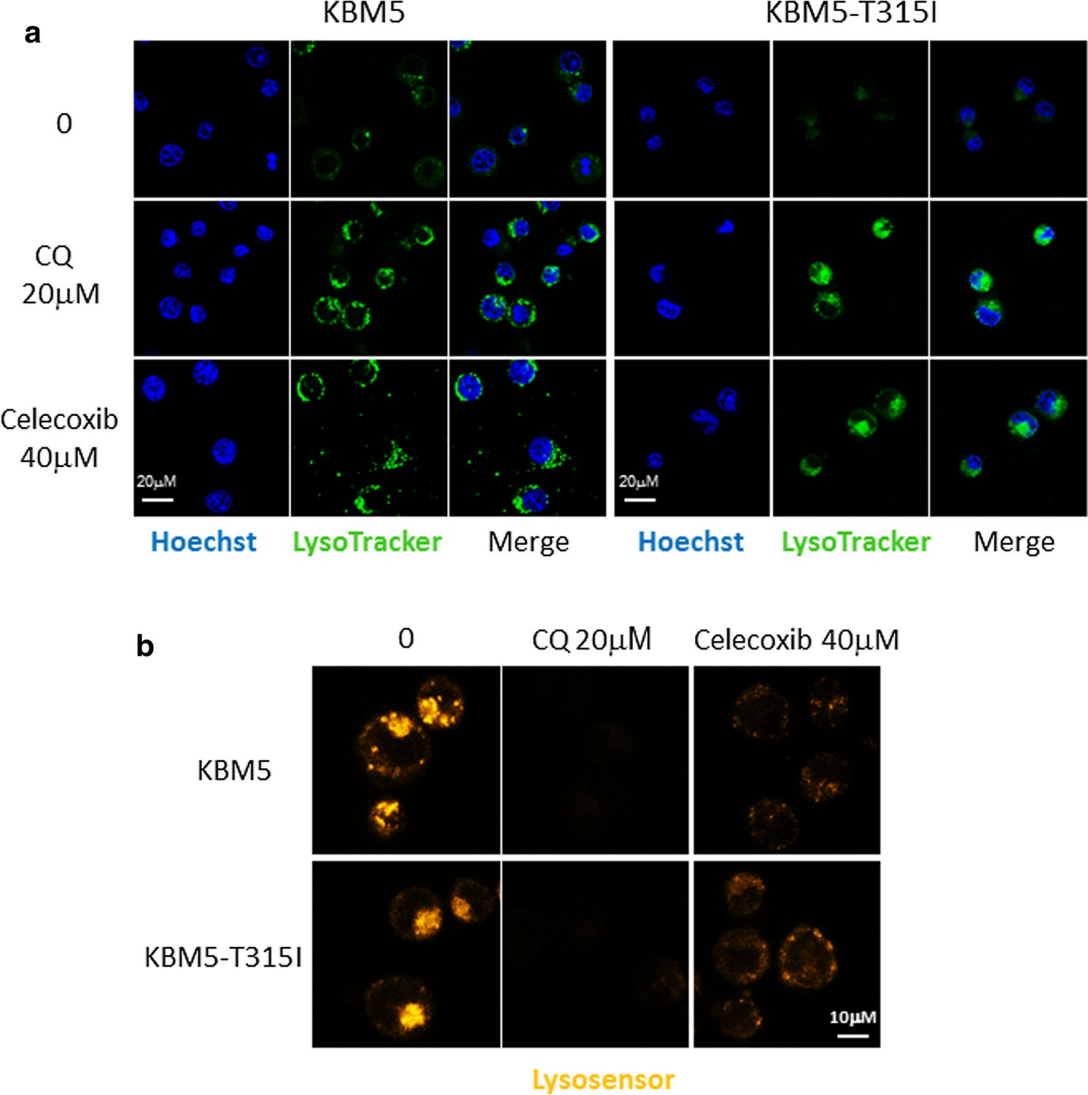
Celecoxib prevents lysosomal function. **a** KBM5 and KBM5-T315I cells were treated with 20 μM CQ or 40 μM celecoxib for 24 h. Hoechst 33342 staining and LysoTracker (Cat. No. L7526) probe labelling assay was used to detect autophagosomes amount and the fluorescence intensity was showed by confocal microscopy. **b** The cells were treated as above, then LysoSensor (Cat. No. L7535) probes labelling assay were conducted to determinate the pH value inside lysosome. The results shown are representative of three independent experiments

The mTOR signal pathway is the most important pathway for autophagy formation [30]. However, there was no change in the expression of phosphorylated mTOR and 4EBP proteins when KBM5 and KMB5-T315I cells were treated with celecoxib (Fig. 5a). Next, the two cell lines were treated with chloroquine (CQ), an autophagy inhibitor which prevents the fusion of autophagosome with lysosome and lysomal protein degradation [31]. The expression of LC3-II proteins was up-regulated in 20 μM CQ treated KBM5 and KMB5-T315I cells (Fig. 5d). CQ and celecoxib had the same effect on the expression of LC3-II. Furthermore, the two cells were treated with CQ combined with celecoxib. Figure 5d displays that LC3-II expression was not increased in cells treated with CQ and celecoxib when compared with cells treated with CQ alone. LysoTracker probes have been used to image acidic spherical organelles [32]. In our study, 40 μM celecoxib or 20 μM CQ treatment both enhanced the green LysoTracker (Cat. No. L7526) fluorescence intensity (Fig. 6a).

### Celecoxib suppresses autophagy by preventing lysosomal functions

Our data above suggest that celecoxib blocks the autophagic flux at the late stage of autophagy. Therefore the effect of celecoxib on lysosomal pH was tested. The LysoSensor reagent exhibits a pH-dependent increase in fluorescence intensity upon acidification [33, 34]. This is in contrast to the LysoTracker probes which exhibit fluorescence that is largely independent of pH. The orange fluorescence intensity of the LysoSensor (Cat. No. L7535) weakened in CML cells treated with 40 μM celecoxib or 20 μM CQ, indicating the neutralization of the intralysosomal pH (Fig. 6b). Then, we stained the two cells with LysoTracker (Cat. No. L7528) and LysoSensor (Cat. No. L7535) simultaneously. As Additional file 1: Figure S1 showed that LysoTracker (Cat. No. L7528) labelled red fluorescence increased as cells treated with celecoxib or CQ, however, the LysoSensor (Cat. No. L7535) labelled green fluorescence was not significantly changed.

### Celecoxib enhances cytotoxicity of imatinib on KBM5-T315I cells

**Fig. 7:**
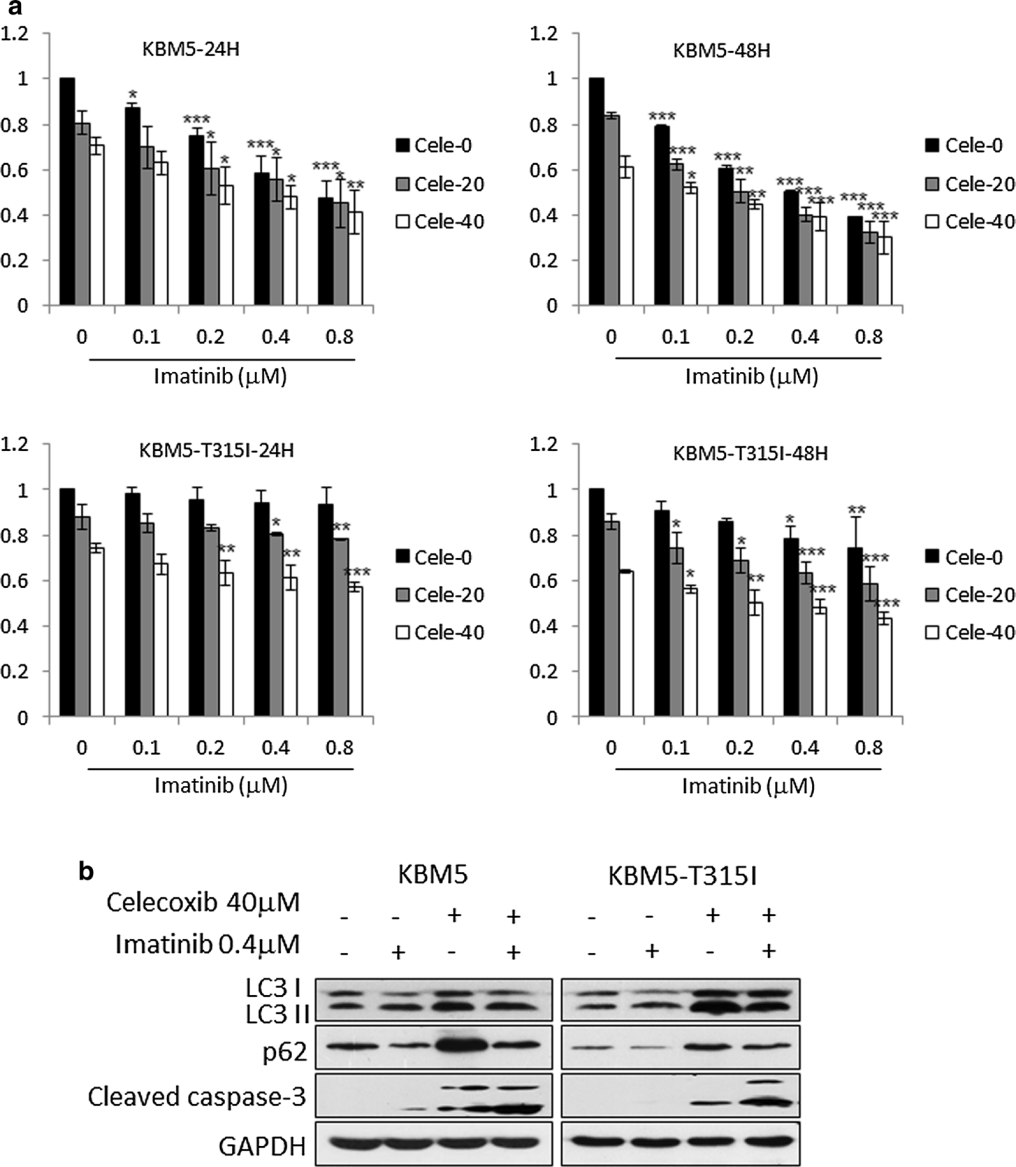
Celecoxib enhances cytotoxicity of imatinib on KBM5 and KBM5-T315I cells. **a** The two cell lines treated with 0, 20, 40 μM celecoxib and 0, 0.1, 0.2, 0.4, 0.8 μM Imatinib for 24 and 48 h were subjected to MTT assay. Data were mean ± SD (n = 3). *p < 0.05, **p < 0.01, ***p < 0.001. **b** The two cell lines were exposed to 40 μM celecoxib and 0.4 μM Imatinib for 24 h and Western blot was conducted for the expression of LC3 and cleaved caspase 3. Three independent experiments were conducted, and representative data were shown

KBM5 and KBM5-T315I cells were treated with celecoxib and imatinib simultaneously and were subsequently detected by the MTT assay to determine the cytostatic efficacy. As shown in Fig. 7a, combination treatment with imatinib and celecoxib had addictive even synergistic cytotoxicity effect in KBM5-T315I cells (Q > 0.85), but not in KBM5 cells. Moreover, autophagy and apoptosis associated proteins were detected. Imatinib increased the ratio of LC3-II to LC3-I protein level and reduced the expression of p62, indicating that autophagy was induced. However, celecoxib could alleviate the effect of imatinib on autophagy by up-regulating p62 expression. Western blot displayed that the expression of cleaved caspase-3 was significantly induced when cells were treated with 40 μM celecoxib combined with 0.4 μM imatinib (Fig. 7b).

## Discussion

Celecoxib is a selective COX-2 inhibitor and its anti-tumor effect has been reported in various cancers [7–9]. In this paper, we demonstrated that the anti-tumor activities of celecoxib included cell cycle arrest, necrosis, apoptosis and autophagy suppression in KBM5 and KBM5-T315I cells. KBM5-T315I cell is a mutation line of KBM5 with a threonine to isoleucine mutation at position 315 in the Abl fragment of the Bcr-Abl kinase domain. This leads to an alteration of the enzymes active site and makes these cells resistant to the first and second generation of TKI [35]. Results showed that celecoxib caused cytotoxic effect in the two CML cell lines which was dose and time-dependent. When extending the celecoxib incubation time, the inhibition effect was stronger in KBM5-T315I cells than in KBM5 cells (Fig. 1), indicating that celecoxib might be used as a new therapeutic agents in imatinib-resistant CML. In accordance with other reports [19, 21], our findings also confirmed that the anti-tumor effect was mediated by cell-cycle arrest at G1 phase (Fig. 3), necrosis and apoptosis induction (Fig. 4) in CML cell lines.

It was interesting to find that celecoxib had an opposite effect on autophagy in CML to that in solid tumors as previously reported. Huang et al. [16] reported that celecoxib induced autophagy in human colon cancer cells and that autophagy inhibitors augmented drug-induced apoptosis. Huang et al. [27] also showed the same results in human urothelial carcinoma cells. However, in these studies, autophagy formation was demonstrated by looking at the LC3-II or Atg12–Atg5 protein levels but not by looking at the levels of the p62 protein. Autophagy is a degradative process that leads to the decomposition of intracellular material in lysosomes [36] and the adaptor proteins p62 and LC3 are the most important markers of this process [23, 24]. The autophagic process can be divided into three steps, the formation of the autophagosomes, the fuse of autophagosomes and lysosomes and the degradation of the autophagic cargo in autolysosomes. Protein p62 can bind to LC3 and ubiquitinated proteins to facilitate autophagic clearance and was degraded in autolysosome [37]. The level of p62 can be regulated by autophagy and accumulates in autophagy deficient cells [38]. Thus, p62 accumulates when autophagy is inhibited and decreased levels can be observed when autophagy takes place. In our study, a variety of approaches were used to determinate the effect of celecoxib on autophagy. Western blot displayed that the ratio of LC3-II to LC3-I and the level of p62 protein were all increased when cells were treated with celecoxib (Fig. 5a, b). The same results were obtained in CML patient samples (Fig. 5c). Given that detection the expression of LC3-II and the fluorescence intensity of LysoTracker probes are measuring different biological events in the autophagic process, they were surprisingly both up-regulated during autophagic process [32, 39]. In our study, celecoxib or CQ treatment both enhanced the LysoTracker fluorescence intensity, indicating an increased autophagosome amount (Fig. 6a). Multiple signal transduction mechanisms are known to regulate autophagy and the mTOR pathway is the most important one for autophagy formation [30]. However, the phosphorylated mTOR and 4EBP proteins, which indicate an activated mTOR pathway, were not changed in the system (Fig. 5a), demonstrating that autophagy was not induced by mTOR pathway. Therefore, these results imply that celecoxib inhibits the autophagic flux at its late stage.

CQ, an inhibitor of autophagy, prevents the fusion between autophagosomes and dilated lysosomes by altering the lysosomal pH [31]. In our study, the effect on autophagy by CQ was the same as the effect by celecoxib indicating that celecoxib worked as an autophagy inhibitor (Fig. 5). Furthermore, the result that celecoxib did not promote the LC3-II level in CML cells which were pretreated with CQ sustained celecoxib to be an autophagy inhibitor (Fig. 5d). To move forward, the weakened fluorescence intensity of the LysoSensor in CML cells treated with celecoxib demonstrated that celecoxib could prevent lysosomal function by altering the lysosomal pH just like CQ to suppress autophagy (Fig. 6b). Furthermore, LysoTraker and LysoSensor dyes were stained simultaneously to CML cells treated with celecoxib or CQ. The result showed that LysoTraker fluorescence increased with LysoSensor fluorescence no significant change which was not consistent with the result of Fig. 6b (Additional file 1: Figure S1). We think the two dyes maybe have interaction effect when stained the CML cells simultaneously, or other unknown mechanisms need further investigation.

Autophagy has been suggested to play a paradoxical role in various cancers. In some types of tumor, autophagy shows a protective role against anticancer treatment, however, others go through autophagic cell death [40, 41]. In hematology, it has become clear that autophagy is one of the survival mechanisms for CML stem cells which are insensitive to all three generations of TKIs [42, 43]. Studies have shown that TKI induced autophagy in CML [44–46]. Sheng et al. [45] showed that Bcr-Abl up-regulated activating transcription factor 5 (ATF5) to stimulate mTORC1 transcription and that imatinib could promote autophagy by inhibition of the PI3 K/Akt/mTORC1 pathway. Meanwhile, Yu et al. [46] showed that imatinib induced autophagy through inhibition of the expression of microRNA-30a and by up-regulation of *BECLIN1* and *ATG5* expression, independent of the mTORC1 pathway. So, it is suggested that imatinib or other chemotherapeutic agents could benefit from the addition of autophagy inhibitors in the treatment of CML. Helgason et al. [44] showed that autophagy inhibitors, such as CQ and 3-MA, could increase CML cell death with anti-leukemia drugs including crotoxin, SAHA, bafetinib, imatinib and dasatinib. In this study, the result showed that celecoxib enhanced the cytotoxicity of imatinib in KBM5-T315I cells but not in KBM5 cells (Fig. 7a). It may because that imatinib itself has good cytotoxicity effect on KBM5 cells, but has only slightly inhibition effect on KBM5-T315I cells. So, when combination of imatinib with celecoxib, the addictive effect was just showed in KBM5-T315I cells. Nevertheless, apoptosis in cells treated with imatinib was enhanced by celecoxib (Fig. 7b). In common with other studies [47–49], imatinib could induce autophagy which was alleviated by celecoxib (Fig. 7b).

## Conclusions

In summary, the present study has demonstrated several features of celecoxib-induced cytotoxicity in both imatinib-sensitive and imatinib-resistant CML cells, namely cell cycle arrest, apoptosis and necrosis induction and autophagy inhibition. It is the first time to reveal that celecoxib acts as an autophagy inhibitor through its effects on the function of lysosomal in CML cell lines. Besides its role in cell cycle arrest and apoptosis induction, celecoxib appears to enhance the cytotoxicity of imatinib in imatinib-resistant CML cells. Thus, the use of celecoxib in combination with imatinib appears to be a promising approach for imatinib-resistant CML therapy, and deserves further investigation.

## Additional file

10.1186/s12967-016-1012-8 LsoTracker and LysoSensor assay. KBM5 and KBM5-T315I cells were treated with 40 μM celecoxib or 20 μM CQ for 24 h. And then the cells were labelled with LsoTracker (Cat. No. L7528) and LysoSensor (Cat. No. L7535) dyes.

## Abbreviations

CML: chronic myelogenous leukemia
TKIs: tyrosine kinase inhibitors
allo-HSCT: allogeneic hematopoietic stem cell transplantation
NSAIDs: non-steroidal anti-inflammatory drugs
COX-2: cyclooxygenase-2
FPA: familial adenomatous polyposis
SDS-PAGE: sodium dodecyl sulfate-polyacrylamide gel electrophoresis
FBS: fetal bovine serum

## References

[1] Rowley JD (1973). Letter: a new consistent chromosomal abnormality in chronic myelogenous leukaemia identified by quinacrine fluorescence and Giemsa staining. Nature.

[2] Groffen J, Stephenson JR, Heisterkamp N, de Klein A, Bartram CR, Grosveld G (1984). Philadelphia chromosomal breakpoints are clustered within a limited region, bcr, on chromosome 22. Cell.

[3] Savage DG, Antman KH (2002). Imatinib mesylate—a new oral targeted therapy. N Engl J Med.

[4] Barrett AJ, Ito S (2015). The role of stem cell transplantation for chronic myelogenous leukemia in the 21st century. Blood.

[5] Gorre ME, Sawyers CL (2002). Molecular mechanisms of resistance to STI571 in chronic myeloid leukemia. Curr Opin Hematol.

[6] Chomel JC, Aggoune D, Sorel N, Turhan AG (2014). Chronic myeloid leukemia stem cells: cross-talk with the niche. Med Sci (Paris).

[7] Johnsen JI, Lindskog M, Ponthan F, Pettersen I, Elfman L, Orrego A, Sveinbjornsson B, Kogner P (2004). Cyclooxygenase-2 is expressed in neuroblastoma, and nonsteroidal anti-inflammatory drugs induce apoptosis and inhibit tumor growth in vivo. Cancer Res.

[8] Hida T, Kozaki K, Muramatsu H, Masuda A, Shimizu S, Mitsudomi T, Sugiura T, Ogawa M, Takahashi T (2000). Cyclooxygenase-2 inhibitor induces apoptosis and enhances cytotoxicity of various anticancer agents in non-small cell lung cancer cell lines. Clin Cancer Res.

[9] Nakata E, Mason KA, Hunter N, Husain A, Raju U, Liao Z, Ang KK, Milas L (2004). Potentiation of tumor response to radiation or chemoradiation by selective cyclooxygenase-2 enzyme inhibitors. Int J Radiat Oncol Biol Phys.

[10] Steinbach G, Lynch PM, Phillips RK, Wallace MH, Hawk E, Gordon GB, Wakabayashi N, Saunders B, Shen Y, Fujimura T (2000). The effect of celecoxib, a cyclooxygenase-2 inhibitor, in familial adenomatous polyposis. N Engl J Med.

[11] Altorki NK, Christos P, Port JL, Lee PC, Mirza F, Spinelli C, Keresztes R, Beneck D, Paul S, Stiles BM (2011). Preoperative taxane-based chemotherapy and celecoxib for carcinoma of the esophagus and gastroesophageal junction: results of a phase 2 trial. J Thorac Oncol.

[12] Tang TC, Poon RT, Lau CP, Xie D, Fan ST (2005). Tumor cyclooxygenase-2 levels correlate with tumor invasiveness in human hepatocellular carcinoma. World J Gastroenterol.

[13] Chow LW, Tung SY, Ng TY, Im SA, Lee MH, Yip AY, Toi M, Gluck S (2013). Concurrent celecoxib with 5-fluorouracil/epirubicin/cyclophosphamide followed by docetaxel for stages II - III invasive breast cancer: the OOTR-N001 study. Expert Opin Invest Drugs.

[14] Altorki NK, Keresztes RS, Port JL, Libby DM, Korst RJ, Flieder DB, Ferrara CA, Yankelevitz DF, Subbaramaiah K, Pasmantier MW, Dannenberg AJ (2003). Celecoxib, a selective cyclo-oxygenase-2 inhibitor, enhances the response to preoperative paclitaxel and carboplatin in early-stage non-small-cell lung cancer. J Clin Oncol.

[15] Mehar A, Macanas-Pirard P, Mizokami A, Takahashi Y, Kass GE, Coley HM (2008). The effects of cyclooxygenase-2 expression in prostate cancer cells: modulation of response to cytotoxic agents. J Pharmacol Exp Ther.

[16] Huang S, Sinicrope FA (2010). Celecoxib-induced apoptosis is enhanced by ABT-737 and by inhibition of autophagy in human colorectal cancer cells. Autophagy.

[17] Kang KB, Zhu C, Yong SK, Gao Q, Wong MC (2009). Enhanced sensitivity of celecoxib in human glioblastoma cells: induction of DNA damage leading to p53-dependent G1 cell cycle arrest and autophagy. Mol Cancer.

[18] Wun T, McKnight H, Tuscano JM (2004). Increased cyclooxygenase-2 (COX-2): a potential role in the pathogenesis of lymphoma. Leuk Res.

[19] Chen C, Xu W, Wang CM (2013). Combination of celecoxib and doxorubicin increases growth inhibition and apoptosis in acute myeloid leukemia cells. Leuk Lymphoma.

[20] Arunasree KM, Roy KR, Anilkumar K, Aparna A, Reddy GV, Reddanna P (2008). Imatinib-resistant K562 cells are more sensitive to celecoxib, a selective COX-2 inhibitor: role of COX-2 and MDR-1. Leuk Res.

[21] Zhang GS, Liu DS, Dai CW, Li RJ (2006). Antitumor effects of celecoxib on K562 leukemia cells are mediated by cell-cycle arrest, caspase-3 activation, and downregulation of Cox-2 expression and are synergistic with hydroxyurea or imatinib. Am J Hematol.

[22] Levine B, Kroemer G (2008). Autophagy in the pathogenesis of disease. Cell.

[23] Nakai A, Yamaguchi O, Takeda T, Higuchi Y, Hikoso S, Taniike M, Omiya S, Mizote I, Matsumura Y, Asahi M (2007). The role of autophagy in cardiomyocytes in the basal state and in response to hemodynamic stress. Nat Med.

[24] Wang QJ, Ding Y, Kohtz DS, Mizushima N, Cristea IM, Rout MP, Chait BT, Zhong Y, Heintz N, Yue Z (2006). Induction of autophagy in axonal dystrophy and degeneration. J Neurosci.

[25] Liu LL, Long ZJ, Wang LX, Zheng FM, Fang ZG, Yan M, Xu DF, Chen JJ, Wang SW, Lin DJ, Liu Q (2013). Inhibition of mTOR pathway sensitizes acute myeloid leukemia cells to aurora inhibitors by suppression of glycolytic metabolism. Mol Cancer Res.

[26] Maiuri MC, Zalckvar E, Kimchi A, Kroemer G (2007). Self-eating and self-killing: crosstalk between autophagy and apoptosis. Nat Rev Mol Cell Biol.

[27] Huang KH, Kuo KL, Ho IL, Chang HC, Chuang YT, Lin WC, Lee PY, Chang SC, Chiang CK, Pu YS (2013). Celecoxib-induced cytotoxic effect is potentiated by inhibition of autophagy in human urothelial carcinoma cells. PLoS One.

[28] Kabeya Y, Mizushima N, Ueno T, Yamamoto A, Kirisako T, Noda T, Kominami E, Ohsumi Y, Yoshimori T (2000). LC3, a mammalian homologue of yeast Apg8p, is localized in autophagosome membranes after processing. EMBO J.

[29] Bjorkoy G, Lamark T, Pankiv S, Overvatn A, Brech A, Johansen T (2009). Monitoring autophagic degradation of p62/SQSTM1. Methods Enzymol.

[30] Jung CH, Ro SH, Cao J, Otto NM, Kim DH (2010). mTOR regulation of autophagy. FEBS Lett.

[31] Yoon YH, Cho KS, Hwang JJ, Lee SJ, Choi JA, Koh JY (2010). Induction of lysosomal dilatation, arrested autophagy, and cell death by chloroquine in cultured ARPE-19 cells. Invest Ophthalmol Vis Sci.

[32] Chikte S, Panchal N, Warnes G (2014). Use of LysoTracker dyes: a flow cytometric study of autophagy. Cytometry A.

[33] Bains M, Heidenreich KA (2009). Live-cell imaging of autophagy induction and autophagosome-lysosome fusion in primary cultured neurons. Methods Enzymol.

[34] You JO, Auguste DT (2010). The effect of swelling and cationic character on gene transfection by pH-sensitive nanocarriers. Biomaterials.

[35] Gibbons DL, Pricl S, Kantarjian H, Cortes J, Quintas-Cardama A (2012). The rise and fall of gatekeeper mutations? The BCR-ABL1 T315I paradigm. Cancer.

[36] Chen N, Karantza V (2011). Autophagy as a therapeutic target in cancer. Cancer Biol Ther.

[37] Pankiv S, Clausen TH, Lamark T, Brech A, Bruun JA, Outzen H, Overvatn A, Bjorkoy G, Johansen T (2007). p62/SQSTM1 binds directly to Atg8/LC3 to facilitate degradation of ubiquitinated protein aggregates by autophagy. J Biol Chem.

[38] Mathew R, Karp CM, Beaudoin B, Vuong N, Chen G, Chen HY, Bray K, Reddy A, Bhanot G, Gelinas C (2009). Autophagy suppresses tumorigenesis through elimination of p62. Cell.

[39] Warnes G (2014). Measurement of autophagy by flow cytometry. Curr Protoc Cytom.

[40] Liu J, Hu XJ, Jin B, Qu XJ, Hou KZ, Liu YP (2012). β-Elemene induces apoptosis as well as protective autophagy in human non-small-cell lung cancer A549 cells. J Pharm Pharmacol.

[41] White E, DiPaola RS (2009). The double-edged sword of autophagy modulation in cancer. Clin Cancer Res.

[42] Graham SM, Jorgensen HG, Allan E, Pearson C, Alcorn MJ, Richmond L, Holyoake TL (2002). Primitive, quiescent, Philadelphia-positive stem cells from patients with chronic myeloid leukemia are insensitive to STI571 in vitro. Blood.

[43] Konig H, Copland M, Chu S, Jove R, Holyoake TL, Bhatia R (2008). Effects of dasatinib on SRC kinase activity and downstream intracellular signaling in primitive chronic myelogenous leukemia hematopoietic cells. Cancer Res.

[44] Helgason GV, Karvela M, Holyoake TL (2011). Kill one bird with two stones: potential efficacy of BCR-ABL and autophagy inhibition in CML. Blood.

[45] Sheng Z, Ma L, Sun JE, Zhu LJ, Green MR (2011). BCR-ABL suppresses autophagy through ATF5-mediated regulation of mTOR transcription. Blood.

[46] Yu Y, Yang L, Zhao M, Zhu S, Kang R, Vernon P, Tang D, Cao L (2012). Targeting microRNA-30a-mediated autophagy enhances imatinib activity against human chronic myeloid leukemia cells. Leukemia.

[47] Salomoni P, Calabretta B (2009). Targeted therapies and autophagy: new insights from chronic myeloid leukemia. Autophagy.

[48] Mishima Y, Terui Y, Mishima Y, Taniyama A, Kuniyoshi R, Takizawa T, Kimura S, Ozawa K, Hatake K (2008). Autophagy and autophagic cell death are next targets for elimination of the resistance to tyrosine kinase inhibitors. Cancer Sci.

[49] Crowley LC, Elzinga BM, O’Sullivan GC, McKenna SL (2011). Autophagy induction by Bcr-Abl-expressing cells facilitates their recovery from a targeted or nontargeted treatment. Am J Hematol.

