# Supplementary figures and images for: Celecoxib suppresses autophagy and enhances cytotoxicity of imatinib in imatinib-resistant chronic myeloid leukemia cells
Source: J Transl Med. 2016 Sep 20;14:270. doi: 10.1186/s12967-016-1012-8 (PMC5029099; doi:10.1186/s12967-016-1012-8)

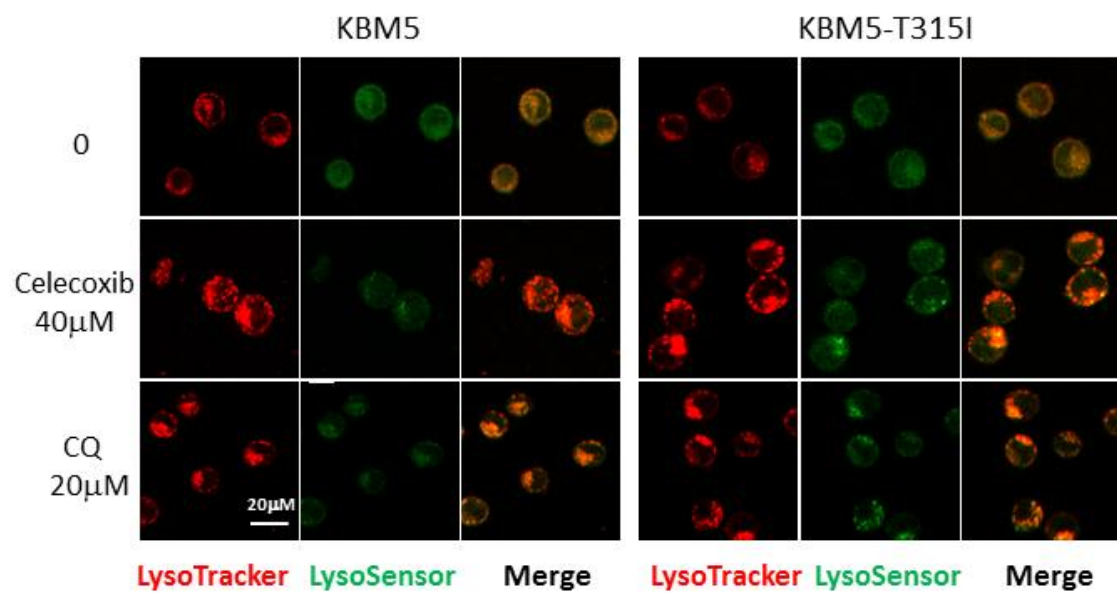

Supplement: Supplementary file 1 — 10.1186/s12967-016-1012-8 LsoTracker and LysoSensor assay. KBM5 and KBM5-T315I cells were treated with 40 μM celecoxib or 20 μM CQ for 24 h. And then the cells were labelled with LsoTracker (Cat. No. L7528) and LysoSensor (Cat. No. L7535) dyes. [file 12967_2016_1012_MOESM1_ESM.pdf]
